# Oral Colonization by *Entamoeba gingivalis* and *Trichomonas tenax*: A PCR-Based Study in Health, Gingivitis, and Periodontitis

**DOI:** 10.3389/fcimb.2021.782805

**Published:** 2021-12-07

**Authors:** Alaa Yaseen, Azmi Mahafzah, Deema Dababseh, Duaa Taim, Ahmad A. Hamdan, Esraa Al-Fraihat, Yazan Hassona, Gülşen Özkaya Şahin, Julien Santi-Rocca, Malik Sallam

**Affiliations:** ^1^ Department of Pathology, Microbiology and Forensic Medicine, School of Medicine, The University of Jordan, Amman, Jordan; ^2^ Department of Clinical Laboratories and Forensic Medicine, Jordan University Hospital, Amman, Jordan; ^3^ School of Dentistry, The University of Jordan, Amman, Jordan; ^4^ Department of Oral and Maxillofacial Surgery, Oral Medicine and Periodontology, Jordan University Hospital, Amman, Jordan; ^5^ Department of Translational Medicine, Faculty of Medicine, Lund University, Malmö, Sweden; ^6^ Department of Clinical Microbiology, Laboratory Medicine, Skåne University Hospital, Lund, Sweden; ^7^ Science and Healthcare for Oral Welfare, Toulouse, France

**Keywords:** periodontopathogens, protozoa, oral microbiota, oral amoebiasis, oral trichomoniasis, *Entamoeba gingivalis*, *Trichomonas tenax*

## Abstract

**Background:**

The etiology of periodontitis remains unclear, as is the place of gingivitis in its pathophysiology. A few studies linked the colonization by oral parasites (*Entamoeba gingivalis* and *Trichomonas tenax*) to periodontal disease and its severity. The aim of the current study was to estimate the prevalence of these oral parasites among healthy individuals, and in patients with gingivitis and periodontitis in Jordan.

**Methods:**

The study was conducted during July 2019–December 2019. Samples were composed of saliva and periodontal material including dental plaque sampled with probes. The detection of oral parasites was done using conventional polymerase chain reaction (PCR).

**Results:**

The total number of study participants was 237: healthy (n=94), gingivitis (n=53) and periodontitis (n=90). The prevalence of *E. gingivalis* was 88.9% among the periodontitis patients, 84.9% among the gingivitis patients and 47.9% in the healthy group. For *T. tenax*, the prevalence was 25.6% among the periodontitis patients, 5.7% among the gingivitis patients and 3.2% in the heathy group. Positivity for *E. gingivalis* was significantly correlated with the presence of periodontal disease compared to the healthy group with odds ratio (OR) of 6.6. Periodontal disease was also correlated with lower monthly income (OR=8.2), lack of dental care (OR=4.8), and history of diabetes mellitus (OR=4.5). Colonization by *E. gingivalis* was correlated with gingivitis (OR=6.1) compared to the healthy group. Colonization by *E. gingivalis* and *T. tenax* were significantly correlated with periodontitis (OR=6.4 for *E. gingivalis*, and OR=4.7, for *T. tenax*) compared to the healthy group. *T. tenax* was only detected among individuals with generalized periodontal disease compared to its total absence among those with localized disease (19.6% vs. 0.0%; p=0.039). The co-infection rate by the two oral parasites was 11.0%.

**Conclusions:**

The higher prevalence of human oral parasites in periodontal disease compared to healthy individuals appears to be more than a mere marker for the disease and might also be associated with disease severity and potential for progression. Thus, the dogmatic view of *E. gingivalis* and *T. tenax* as commensals needs to be re-evaluated and their contribution to pathophysiology of periodontal diseases cannot be neglected.

## Introduction

The role of microbiota in health and disease among humans has recently been demonstrated using molecular methods (Cho and Blaser, 2012; Siqueira and Rocas, 2017; Tsuji et al., 2018). The oral microbiota was no exception, and its disruption has been linked to a various range of oral diseases including gingivitis and periodontitis (Lourenco et al., 2014; Lamont et al., 2018; Santi-Rocca, 2020).

The complex interactions of different resident microbes that result in an equilibrium to maintain the healthy state of the oral cavity is termed oral eubiosis (Lamont et al., 2018). In contrast to eubiosis, the disruption in oral microbiota’s homeostatic state is referred to as oral dysbiosis (Darveau, 2010; Lourenco et al., 2014; Kinane et al., 2017).

Periodontal disease represents a state of chronic inflammation in gingiva, bone and supporting ligaments, with gingivitis and periodontitis as the most common presentations (Di Benedetto et al., 2013; Kononen et al., 2019). The physiologic healthy state of gingiva can be defined as the total absence or minimal levels of clinical inflammation of the periodontium with normal support (no loss affecting attachment or bone) (Caton et al., 2018; Lang and Bartold, 2018). The identification of plaque‐induced gingivitis relies on the presence of bleeding on probing with an intact periodontium and/or visible inflammation; and this condition can be reversed back to a healthy state if managed properly (Caton et al., 2018; Trombelli et al., 2018). Periodontitis results in the destruction of periodontal ligament, cementum and alveolar bone, as well as migration of the long junctional epithelium. The inflammation and microbiota of periodontitis can be controlled; however, the tissues are not healed back to their initial volume, organization, and shape (Caton et al., 2018). Thus, continual maintenance of good oral hygiene is a necessity in such case (American Academy of Periodontology, 2011; Zimmermann et al., 2015; Lertpimonchai et al., 2017).

Periodontal disease is considered among the most common diseases affecting all age groups with predilection for the elderly (Kinane et al., 2017; Tonetti et al., 2017). As of 2010, the prevalence of periodontitis was 47% among adults aged 30 and above in the United States, while the global prevalence of severe periodontitis was 11%, with higher estimates for gingivitis (Eke et al., 2012; Jin et al., 2016; Nazir, 2017). In addition, periodontitis is considered an important cause of tooth loss in older adults, which adversely affects the quality of life among this group (Griffin et al., 2012). A recent study estimated the prevalence of periodontitis among dentate US adults aged 30 years or more at 42%, with 7.8% having severe form of the disease (Eke et al., 2020).

The underlying etiology and pathogenesis of periodontal disease has been linked to microbial dysbiosis (Mira et al., 2017; Sudhakara et al., 2018). However, the exact specific roles of different microbes in the dental plaque that could lead to the development of periodontal disease remains an enigma (Darveau, 2010; Hajishengallis, 2015). In addition, the initiating factors for microbial dysbiosis in the oral cavity remains unclear and deciphering such factors is the subject for ongoing research (Lamont et al., 2018).

Several microbiological patterns can be identified in periodontal diseases, in association with some specific pathophysiological traits, sustaining that the diversity in periodontitis is not limited to the variety of the current consensual classification of clinical presentations (Offenbacher et al., 2016). However, the presence of inflammation-related bone destruction is a common defining characteristic of periodontitis (Cekici et al., 2014). Thus, deep understanding of the inflammatory and immunologic processes observed in periodontal disease is of prime importance in any attempt for management of such a highly prevalent disease (Kononen et al., 2019).

Several risk factors have been linked to an increased incidence of periodontal disease; and these can be divided into non-modifiable and modifiable factors (Van Dyke and Sheilesh, 2005). Examples of non-modifiable factors include aging, genetic predisposition, and osteoporosis; while modifiable factors include smoking, diabetes mellitus, psychological stress, alcohol consumption and poor oral hygiene (Van Dyke and Sheilesh, 2005; Borojevic, 2012; Reynolds, 2014; Hong et al., 2016; Wang and McCauley, 2016; Koo and Hong, 2018; Liu et al., 2018; Masumoto et al., 2019).

The role of the yet-identified parasitic fraction of the oral microbiome, namely: *Entamoeba gingivalis* and *Trichomonas tenax*, is gaining interest as potential contributing factors to the development of periodontal disease (Marty et al., 2017; Bonner et al., 2018; Eslahi et al., 2021). Several studies aimed to investigate oral colonization by these parasites among healthy individuals and those with periodontal disease with variable results (Athari et al., 2007; Ghabanchi et al., 2010; Al-hamiary et al., 2011; Trim et al., 2011; Ibrahim and Abbas, 2012; Bonner et al., 2014; Yazar et al., 2016; Hassan et al., 2019). Such variability can be related to adoption of different approaches for parasite detection, and the existence of previously unknown genetic variants of oral parasites (Garcia et al., 2018b; Santi-Rocca, 2020). In addition, variability in the prevalence of oral parasites in health and disease can be attributed to limitations of small sample sizes, and possible bias in selection of study subjects among others as reviewed recently by (Santi-Rocca, 2020).

The objective of the current study was to investigate the prevalence of *E. gingivalis* and *T. tenax* in health and periodontal disease. Also, we aimed to better define the place of gingivitis in the physiopathology of periodontal disease using parasite colonization. Finally, we aimed to identify the variables that might be associated with increased likelihood of harboring these oral parasites in health and disease.

## Materials and Methods

### Study Design

The prospective study with active enrolment of potential participants was carried out at Jordan University Hospital (JUH), Amman, Jordan from July to December 2019.

We sought to recruit study subjects from the following three categories: (1) Individuals with healthy gingiva (will be referred to as “healthy group” in the rest of manuscript). This healthy group was defined based on a healthy periodontium with no attachment loss, no bleeding upon probing (BOP) or minimal BOP (<10%), and no anatomical loss of periodontal structures, with absence of clinical signs of inflammation; (2) Individuals with gingivitis (herein, the term “gingivitis” will be applied to plaque‐induced gingivitis, rather than non‐dental‐biofilm induced forms of gingivitis); and (3) individuals with periodontitis.

The individuals with gingivitis and those with periodontitis -which represented the “disease group”- were recruited from Periodontics Outpatient Clinics at JUH using a convenience sampling approach, whereas the healthy controls were recruited by active approach of the JUH staff that included dentists, laboratory technicians, nurses and students at the University of Jordan.

### Ethical Statement

This study was approved by the School of Medicine and the School of Graduate Studies, University of Jordan. Ethical approval was obtained from the Institutional Review Board (IRB) at JUH (Ref. No. 239/2019).

A written and signed informed consent was obtained from all individuals who agreed to participate in the study following full explanation of the study objectives and the procedure of obtaining the samples (**Supplementary Material**). In addition, the work was conducted according to the principles of good clinical practice that have their origin in the declaration of Helsinki and all individual data were treated with confidentiality.

### Eligibility Criteria for Participation in the Study

Each healthy individual was included in the study if the following criteria were met altogether: 1) Healthy gingiva on periodontal examination; 2) Bleeding index (BI) of less than 10%; and 3) No previous history of periodontal diseases. The BI was calculated as follows: six representative teeth from all quadrants were chosen, and each tooth was gently probed with a University of North Carolina (UNC) periodontal probe (15 mm) at four sites (mesial, mid-buccal, mid-lingual, and distal). A dichotomous reading was used where bleeding is scored as present (given a score of 1) or absent (given a score of 0) and the number of sites where bleeding is present was recorded. The BI as a percentage was then defined through dividing the number of sites where bleeding was recorded by the total number of sites tested multiplied by 100. A controlled gentle probing force [well-tolerated by the patient (25 g)] was used (Newbrun, 1996).

The inclusion criteria for individuals with gingivitis and periodontitis were: 1) Diagnosis of the periodontal disease for the first time; and 2) No previous history of exposure to any kind of periodontal therapy (scaling or root planing).

The presence of one of the following criteria resulted in exclusion of the potential participant from the study: 1) Pregnancy; 2) Previous history of periodontal treatment; 3) Non‐dental biofilm-induced forms of gingivitis; 4) Presence of dental implants; 5) Recent use of antibiotics; or 6) Orthodontics treatment.

### Assessment of the Possible Risk Factors for Periodontal Disease

Data from the study participants were collected using a paper-based questionnaire (**Supplementary Material**). The study participants’ data included: age, gender, nationality, body mass index (BMI), monthly income, dental care level, history of smoking, history of alcohol consumption, history of diabetes mellitus (DM), family history of gingival disease and history of osteoporosis.

In addition, nine questions (adopted from the Perceived Stress Scale) to assess the stress-related factors, with each positive answer given a single point yielding a stress score that ranged from nil to nine (Nielsen et al., 2016). The study population was divided into two groups based on stress score as follows: “lower stress group” with a stress score of zero to 4, and “higher stress group” with a stress score of 5 to 9. The Cronbach’s α value of 0.855 indicated an acceptable internal consistency for the proposed stress scale in this study.

### Classification of the Study Subjects

The diagnosis of gingivitis and periodontitis was based on diagnostic guidelines that were set by the 2018 new classification scheme for periodontal and peri-implant diseases and conditions (Caton et al., 2018).

The detailed approach of evaluating the study subjects is provided in (**Supplementary Material**). Bleeding on probing and supragingival plaque presence (1) or absence (0) were evaluated in 4 sites of 6 teeth (details in **Supplementary Material**). The mean of the 24 sites gave the bleeding index (BI) and the plaque index (PI), expressed as percentages. In addition, the periodontal screening and recording (PSR) score was evaluated (details in **Supplementary Material**) (Caton et al., 2018).

The study subject was classified into the “healthy group” based on a BI < 10% and periodontal screening and recording (PSR) score of zero. The sample from each healthy participant was composed of a sub-gingival plaque along with saliva.

If the BI was ≥ 10% and ≤ 30%, then the participant was considered to have a “localized gingivitis”. The study subjects with BI > 30% were considered to have a “generalized gingivitis”.

Study subjects were classified into the “periodontitis group” after full periodontal examination if there was interdental clinical attachment loss detectable at two or more non-adjacent teeth, or buccal or oral clinical attachment loss (CAL) ≥ 2 mm with pocket depth > 3mm detectable at two or more teeth. Once the patient was diagnosed with periodontitis, staging and grading were done according to the recent classification of 2018 (Caton et al., 2018; Papapanou et al., 2018).

### Specimen Collection

Salivary and dental plaque specimens were obtained from each study subject. Supra-gingival plaque was removed then the sample was taken from the deepest periodontal pocket. Dental plaque samples were collected using the UNC periodontal probe (15 mm). For participants with furcation involvement, the sample was taken using Naber’s probe (Nordent manufacturing Inc., Illinois, the United States) from the furcation. For each participant, the salivary and dental plaque specimens were mixed together in a sterile tube that was stored at −20°C for DNA extraction and amplification.

### DNA Purification and Amplification

Purification of DNA from the saliva/dental plaque specimens was done using QIAamp DNA Mini Kit (QIAGEN, Hilden, Germany) according to manufacturer’s instructions (**Supplementary Material**).

For the detection of oral parasites by PCR, two sets of PCR primers were used. For *E. gingivalis* ST1, we used the same set of primers utilized by Bonner et al. with a minor modification of the reverse primer as follows: forward primer (5′-AGGAATGAACGGAACGTACA-3′) and reverse primer (5′-CCATTTCCTTCTTCTATTGTTTMAC-3′) with a product size of 203 bases in the 18S ribosomal RNA region (Bonner et al., 2014).

For *T. tenax*, we used the same set of primers utilized by Kikuta et al. as follows: PT3 forward primer (5′-AGTTCCATCGATGCCATTC-3′) and PT7 reverse primer (5′-GCATCTAAGGACTTAGACG-3′) with product size of 776 bases in 18S ribosomal RNA region (Kikuta et al., 1997).

The PCR mix comprised 5 μL of the DNA eluate, 5 μL of 5×FIREPol Master Mix (Solis BioDyne), 1 μL of each primer and 13 μL of DNase/RNase free water. The steps of PCR were as follows: Initial denaturation for 3.5 minutes at 94°C, 40 cycles of 1 minute at 94°C for denaturation, 1 minute at 60°C for primer annealing, 1 minute at 72°C for elongation, a final elongation step for 5 minutes at 72°C (Kikuta et al., 1997; Kucknoor et al., 2009; Bonner et al., 2014).

Proper positive and negative controls (patient samples with motile *Entamoeba* and *Trichomonas* that yielded the expected amplicon sizes as positive controls, and nuclease-free water as the negative control) for purification and PCR were used to ensure the quality of DNA extraction and PCR and to rule out contamination. The housekeeping gene actin beta (*ACTB*) with accession number (NG_007992) was used to assess PCR inhibition of the sample and to ensure the efficiency of the DNA purification procedure with the following primers: forward 5’-GTCCTGTGGCATCCACGAAA-3’ and reverse 5’-AGTGAGGACCCTGGATGTGAC-3’ and the PCR product size was 265 bases.

### Statistical Analysis

Data generated from the study were edited using Microsoft Excel and uploaded to IBM SPSS Statistics 22.0 for Windows (Armonk, New York, the United States: IBM Corp). Two-sided Fisher’s exact test (FET), Chi-squared test (χ^2^ test), Mann-Whitney *U* (M-W), Kruskal-Wallis (K-W) and linear-by-linear test for association (LBL) tests were used when appropriate and the statistical significance was considered for p ≤ 0.050.

To analyse the patterns associated with higher likelihood of having periodontal disease as a whole and per disease state (gingivitis and periodontitis), we conducted multinomial logistic regression analysis using variables that were classified into dichotomous outcomes. Confidence intervals of percentages (95% CI) were calculated using modified Wald method through GraphPad calculator available freely online through the following link: https://www.graphpad.com/quickcalcs/ConfInterval1.cfm

Sample size determination was based on calculations done *via* Epitools - Epidemiological Calculators available freely online (https://epitools.ausvet.com.au/). The minimum number of participants in each study group was determined at 53 based on the following parameters in “Sample size for a Case-control study”: Expected proportion in controls=0.05, assumed odds ratio=5.00, confidence level=0.90, and power=0.80

## Results

### Characteristics of the Study Population

The total number of study participants who were eligible to be included in final analysis was 237, distributed as follows: healthy group (n=94, 39.7%), gingivitis group (n=53, 22.4%) and periodontitis group (n=90, 38.0%, **Table 1**).

**Table 1:** Characteristics of the study population divided by the three study groups.

Characteristic	*Category*	Study group			P-value^9^
		Healthy N (%)	Gingivitis N (%)	Periodontitis N (%)	
Age in years (mean, SD^1^)*		33 (13.5)	37 (14.5)	46 (10.9)	<0.001
Stress scale^2^ (mean, SD)		2.9 (2.5)	4.5 (2.7)	5.7 (2.7)	<0.001
Sex	*Male*	40 (42.6)	29 (54.7)	53 (58.9)	0.074
	*Female*	54 (57.4)	24 (45.3)	37 (41.1)	
Nationality	*Jordanian*	88 (93.6)	48 (90.6)	86 (95.6)	0.496
	*Non-Jordanian*	6 (6.4)	5 (9.4)	4 (4.4)	
BMI^3^	*≤ 25*	50 (53.8)	27 (50.9)	28 (31.5)	0.006
	*> 25*	43 (46.2)	26 (49.1)	61 (68.5)	
Monthly income	*≤ 1000 JOD^7^*	51 (54.3)	46 (88.5)	89 (98.9)	<0.001
	*> 1000 JOD*	43 (45.7)	6 (11.5)	1 (1.1)	
Dental care level	*Any form of care^8^*	71 (75.5)	15 (28.8)	19 (21.1)	<0.001
	*None*	23 (24.5)	37 (71.2)	71 (78.9)	
DM^4^	*No*	80 (85.1)	50 (94.3)	76 (84.4)	0.190
	*Yes*	14 (14.9)	3 (5.7)	14 (15.6)	
Family history^5^	*No*	69 (78.4)	39 (79.6)	58 (69.9)	0.323
	*Yes*	19 (21.6)	10 (20.4)	25 (30.1)	
Smoking^6^	*Never*	63 (67.0)	26 (49.1)	42 (46.7)	0.012
	*Current/ex-smoker*	31 (33.0)	27 (50.9)	48 (53.3)	
Alcohol use	*Never*	89 (94.7)	51 (96.2)	86 (95.6)	0.907
	*Current/former use*	5 (5.3)	2 (3.8)	4 (4.4)	
Osteoporosis	*No*	88 (95.7)	46 (88.5)	79 (91.9)	0.270
	*Yes*	4 (4.3)	6 (11.5)	7 (8.1)	

^1^SD, Standard deviation; ^2^Stress scale, Nine-item based scale that was adopted from the Perceived Stress Scale, to assess the stress-related factors with a range (0-9); ^3^BMI, Body mass index; ^4^DM, History of diabetes mellitus; ^5^Family history, Previous diagnosis of periodontal disease in a family member; ^6^Smoking, Includes cigarettes, e-cigarettes, pipe, shisha, and narghile; ^7^JOD, Jordanian dinar; ^8^Any form of care, Any form of dental care including annual inspection, regular and irregular monitoring and cleaning; ^9^P-value, Calculated using chi-squared and Kruskal Wallis tests; *Number of missing information were as follows: for age (n=1) among the periodontitis group.

Significant differences among the three study groups were found for the following factors: the median age of the healthy group was younger compared to the disease group (24 vs. 44 years; p<0.001; M-W). The periodontitis group had an older median age compared to the two other groups (p<0.001; K-W, **Table 1**).

Additional differences between the three study groups were noticed as follows: higher BMI, lower monthly income, lack of dental care, and current/previous history of smoking were found for the periodontitis group (**Table 1**).

### The Prevalence of Oral Parasites in the Whole Study Population

The PCR testing was performed for all study subjects (n=237). The overall prevalence of *E. gingivalis* among the study subjects was 71.7% (95% CI: 65.7% to 77.1%), while the overall prevalence for *T. tenax* was 12.2% (95% CI: 8.6% to 17.1%).

**Figure 1:**
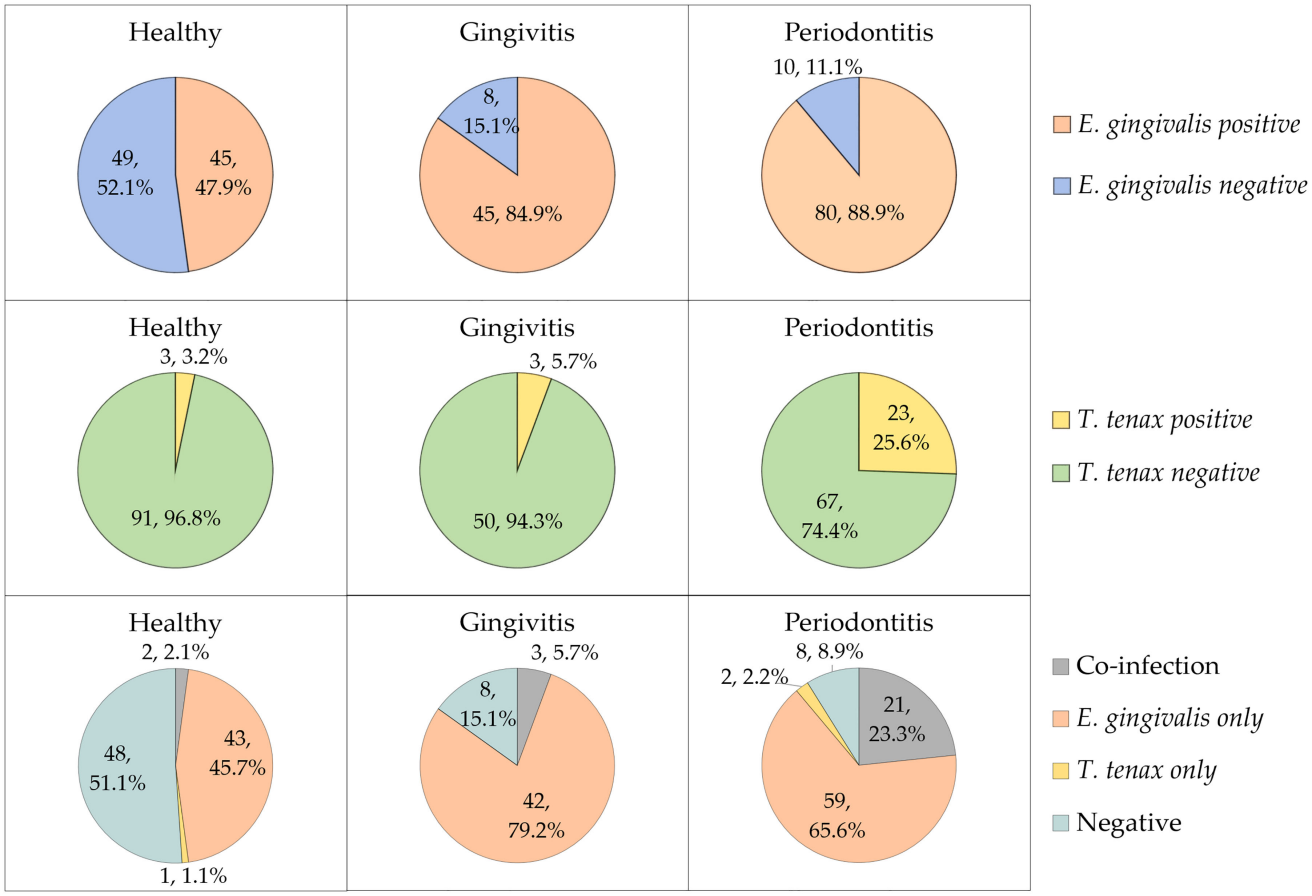
The prevalence of *Entamoeba gingivalis* and *Trichomonas tenax* in the study participants divided by study groups (health, gingivitis and periodontitis). Co-infection denoted the concurrent detection of *E. gingivalis* and *T. tenax*.

Stratified by the three study groups, the prevalence of *E. gingivalis* was the highest among the periodontitis group (n=80/90, 88.9%), compared to the gingivitis group (n=45/53, 84.9%), while the healthy group had the lowest prevalence (n=45/94, 47.9%, **Figure 1**). The difference in *E. gingivalis* prevalence was statistically significant upon comparing the healthy group to the gingivitis and periodontitis groups (p<0.001 for both comparisons; χ^2^ test). However, the difference was not statistically significant upon comparing the gingivitis group with the periodontitis group (p=0.603; χ^2^ test).

For *T. tenax*, the prevalence increased starting from 3.2% in the heathy group, to 5.7% in the gingivitis group and reaching 25.6% in the periodontitis group (**Figure 1**). The difference lacked statistical significance upon comparing the healthy and gingivitis groups (p=0.668; χ^2^ test). However, a statistically significant result was noticed upon comparing the periodontitis group to healthy and gingivitis groups (p<0.001 and p=0.003 respectively for the two comparisons; χ^2^ test).

Concurrent detection of the two oral parasites (dual colonization) was found in 26 study subjects yielding a prevalence of 11.0% (95% CI: 7.6% to 15.6%). Among the 29 study subjects with *T. tenax* colonization, *E. gingivalis* was also detected in 26 individuals (89.7%).

### Factors Associated With a Higher Prevalence of Oral Parasites in Each Study Group

We aimed to seek the possible factors associated with a higher prevalence of *E. gingivalis*. However, as some parameters from these populations were impacted by our recruitment, as previously cited, we chose to perform this analysis without merging the three study groups. A higher prevalence of *E. gingivalis* in the healthy group was found among participants with a history of DM compared to those who did not have the disease (78.6% vs. 42.5%; p=0.019, FET, **Table 2**). For the other tested variables, the prevalence of *E. gingivalis* in each study group did not show statistically significant differences (**Table 2**).

**Table 2:** Factors associated with a higher prevalence of *Entamoeba gingivalis* stratified per study group.

Characteristic	Study group	Healthy		P-value^9^	Gingivitis		P-value	Periodontitis		P-value
	*E. gingivalis*	Positive N^8^ (%)	Negative N (%)		Positive N (%)	Negative N (%)		Positive N (%)	Negative N (%)	
**Age group**	*≤ 38*	33 (49.3)	34 (50.7)	0.820	28 (84.8)	5 (15.2)	1.000	16 (80.0)	4 (20.0)	0.223
	*> 38*	12 (44.4)	15 (55.6)		17 (85.0)	3 (15.0)		63 (91.3)	6 (8.7)	
**Sex**	*Male*	21 (52.5)	19 (47.5)	0.532	23 (79.3)	6 (20.7)	0.269	47 (88.7)	6 (11.3)	1.000
	*Female*	24 (44.4)	30 (55.6)		22 (91.7)	2 (8.3)		33 (89.2)	4 (10.8)	
**Nationality**	*Jordanian*	41 (46.6)	47 (53.4)	0.421	40 (83.3)	8 (16.7)	1.000	76 (88.4)	10 (11.6)	1.000
	*Non-Jordanian*	4 (66.7)	2 (33.3)		5 (100)	0 (0)		4 (100)	0 (0)	
**BMI^1^**	*≤ 25*	26 (52.0)	24 (48.0)	0.534	24 (88.9)	3 (11.1)	0.467	22 (78.6)	6 (21.4)	0.066
	*> 25*	19 (44.2)	24 (55.8)		21 (80.8)	5 (19.2)		57 (93.4)	4 (6.6)	
**Monthly income**	*≤ 1000 JOD^6^*	27 (52.9)	24 (47.1)	0.307	40 (87.0)	6 (13.0)	0.227	79 (88.8)	10 (11.2)	1.000
	*> 1000 JOD*	18 (41.9)	25 (58.1)		4 (66.7)	2 (33.3)		1 (100)	0 (0)	
**Dental care**	*Any form of care^7^*	31 (43.7)	40 (56.3)	0.230	13 (86.7)	2 (13.3)	1.000	17 (89.5)	2 (10.5)	1.000
	*None*	14 (60.9)	9 (39.1)		31 (83.8)	6 (16.2)		63 (88.7)	8 (11.3)	
**Smoking^2^**	*Never*	29 (46.0)	34 (54.0)	0.664	23 (88.5)	3 (11.5)	0.704	36 (85.7)	6 (14.3)	0.505
	*Current/ex-smoker*	16 (51.6)	15 (48.4)		22 (81.5)	5 (18.5)		44 (91.7)	4 (8.3)	
**DM^3^**	*No*	34 (42.5)	46 (57.5)	0.019	44 (88.0)	6 (12.0)	0.056	67 (88.2)	9 (11.8)	1.000
	*Yes*	11 (78.6)	3 (21.4)		1 (33.3)	2 (66.7)		13 (92.9)	1 (7.1)	
**Family history^4^**	*Yes*	8 (42.1)	11 (57.9)	0.607	10 (100)	0 (0)	0.319	23 (92.0)	2 (8.0)	0.716
	*No*	35 (50.7)	34 (49.3)		32 (82.1)	7 (17.9)		50 (86.2)	8 (13.8)	
**Alcohol use**	*Never*	41 (46.1)	48 (53.9)	0.190	43 (84.3)	8 (15.7)	1.000	76 (88.4)	10 (11.6)	1.000
	*Current/former use*	4 (80.0)	1 (20.0)		2 (100)	0 (0)		4 (100)	0 (0)	
**Osteoporosis**	*Yes*	1 (25.0)	3 (75.0)	0.618	5 (83.3)	1 (16.7)	1.000	7 (100)	0 (0)	1.000
	*No*	43 (48.9)	45 (51.1)		39 (84.8)	7 (15.2)		69 (87.3)	10 (12.7)	
**Stress score^5^**	*Less stressed*	37 (49.3)	38 (50.7)	0.616	30 (88.2)	4 (11.8)	0.436	38 (90.5)	4 (9.5)	0.745
	*More stressed*	8 (42.1)	11 (57.9)		15 (78.9)	4 (21.1)		42 (87.5)	6 (12.5)	

^1^BMI, Body mass index; ^2^Smoking, Includes cigarettes, e-cigarettes, pipe, shisha, and narghile; ^3^DM, History of diabetes mellitus; ^4^Family history, Previous diagnosis of periodontal disease in a family member; ^5^Stress scale, Nine-item based scale that was adopted from the Perceived Stress Scale, to assess the stress-related factors with a range (0-9); ^6^JOD, Jordanian dinar; ^7^Any form of care, Any form of dental care including annual inspection, regular and irregular monitoring and cleaning; ^8^N, Number; ^9^P-value, Calculated using two-sided Fisher’s exact test.

For *T. tenax*, a higher prevalence was found in the periodontitis group among non-Jordanians (100.0% vs. 22.1%; p=0.003, FET), and among periodontitis patients with a BMI > 25 (32.8% vs. 10.7%; p=0.036, FET). In the gingivitis group, participants with a higher level of stress had a higher prevalence of *T. tenax* (15.8%) compared to its total absence among the less stressed participants in this group (p=0.041, FET). For the other tested variables, a lack of statistically significant differences in the prevalence of *T. tenax* within each study group (healthy, gingivitis and periodontitis) was noticed (**Table 3**).

**Table 3:** Factors associated with a higher prevalence of *Trichomonas tenax* stratified per study group.

Characteristic	Study group	Healthy		P-value^9^	Gingivitis		P-value	Periodontitis		P-value
	*T. tenax*	Positive N^8^ (%)	Negative N (%)		Positive N (%)	Negative N (%)		Positive N (%)	Negative N (%)	
**Age group**	*≤ 38*	2 (3.0)	65 (97.0)	1.000	1 (3.0)	32 (97.0)	0.549	6 (30.0)	14 (70.0)	0.772
	*> 38*	1 (3.7)	26 (96.3)		2 (10.0)	18 (90.0)		17 (24.6)	52 (75.4)	
**Sex**	*Male*	2 (5.0)	38 (95.0)	0.573	2 (6.9)	27 (93.1)	1.000	16 (30.2)	37 (69.8)	0.326
	*Female*	1 (1.9)	53 (98.1)		1 (4.2)	23 (95.8)		7 (18.9)	30 (81.1)	
**Nationality**	*Jordanian*	3 (3.4)	85 (96.6)	1.000	2 (4.2)	46 (95.8)	0.262	19 (22.1)	67 (77.9)	0.003
	*Non-Jordanian*	0 (0)	6 (100)		1 (20.0)	4 (80.0)		4 (100)	0 (0)	
**BMI^1^**	*≤ 25*	1 (2.0)	49 (98.0)	0.594	1 (3.7)	26 (96.3)	0.610	3 (10.7)	25 (89.3)	0.036
	*> 25*	2 (4.7)	41 (95.3)		2 (7.7)	24 (92.3)		20 (32.8)	41 (67.2)	
**Monthly income**	*≤ 1000 JOD^6^*	3 (5.9)	48 (94.1)	0.247	3 (6.5)	43 (93.5)	1.000	23 (25.8)	66 (74.2)	1.000
	*> 1000 JOD*	0 (0)	43 (100)		0 (0)	6 (100)		0 (0)	1 (100)	
**Dental care**	*Any form of care^7^*	2 (2.8)	69 (97.2)	1.000	0 (0)	15 (100)	0.548	6 (31.6)	13 (68.4)	0.557
	*None*	1 (4.3)	22 (95.7)		3 (8.1)	34 (91.9)		17 (23.9)	54 (76.1)	
**Smoking^2^**	*Never*	2 (3.2)	61 (96.8)	1.000	0 (0)	26 (100)	0.236	13 (31.0)	29 (69.0)	0.336
	*Current/ex-smoker*	1 (3.2)	30 (96.8)		3 (11.1)	24 (88.9)		10 (20.8)	38 (79.2)	
**DM^3^**	*No*	3 (3.8)	77 (96.3)	1.000	3 (6.0)	47 (94.0)	1.000	22 (28.9)	54 (71.1)	0.105
	*Yes*	0 (0)	14 (100)		0 (0)	3 (100)		1 (7.1)	13 (92.9)	
**Family history^4^**	*Yes*	2 (10.5)	17 (89.5)	0.116	1 (10.0)	9 (90.0)	0.504	3 (12.0)	22 (88.0)	0.060
	*No*	1 (1.4)	68 (98.6)		2 (5.1)	37 (94.9)		19 (32.8)	39 (67.2)	
**Alcohol use**	*Never*	3 (3.4)	86 (96.6)	1.000	2 (3.9)	49 (96.1)	0.111	22 (25.6)	64 (74.4)	1.000
	*Current/former use*	0 (0)	5 (100)		1 (50.0)	1 (50.0)		1 (25.0)	3 (75.0)	
**Osteoporosis**	*Yes*	0 (0)	4 (100)	1.000	0 (0)	6 (100)	1.000	1 (14.3)	6 (85.7)	0.669
	*No*	3 (3.4)	85 (96.6)		3 (6.5)	43 (93.5)		22 (27.8)	57 (72.2)	
**Stress score^5^**	*Less stressed*	3 (4.0)	72 (96.0)	1.000	0 (0)	34 (100)	0.041	14 (33.3)	28 (66.7)	0.148
	*More stressed*	0 (0)	19 (100)		3 (15.8)	16 (84.2)		9 (18.8)	39 (81.3)	

^1^BMI, Body mass index; ^2^Smoking, Includes cigarettes, e-cigarettes, pipe, shisha, and narghile; ^3^DM, History of diabetes mellitus; ^4^Family history, Previous diagnosis of periodontal disease in a family member; ^5^Stress scale, Nine-item based scale that was adopted from the Perceived Stress Scale, to assess the stress-related factors with a range (0-9); ^6^JOD, Jordanian dinar; ^7^Any form of care, Any form of dental care including annual inspection, regular and irregular monitoring and cleaning; ^8^N, Number; ^9^P-value, Calculated using two-sided Fisher’s exact test.

### Risk Factors for Periodontal Disease

The majority of risk factors for periodontal disease that were previously reported in various studies were tested in this work (e.g. dental care level, smoking, DM, etc.). To analyse the patterns associated with higher likelihood of having periodontal disease as a whole and per disease state (gingivitis and periodontitis), we conducted multinomial logistic regression analysis using the following variables as covariates that were classified into dichotomous outcomes as follows: age [> 38 years vs. ≤ 38 years, (38 years was the median age for the whole population)], sex (male vs. female), nationality (Jordanian vs. non-Jordanian), BMI (> 25 vs. ≤ 25), monthly income (≤ 1000 JOD vs. > 1000 JOD), dental care (no dental care vs. any form of dental care), smoking (current/ex-smoker vs. non-smoker), DM vs. non-diabetic, family history (present vs. absent), subjective evaluation of stress (more stressed if the score is 0-4 vs. less stressed if the score is 5-9), alcohol use (current/ex-user vs. non-consumer), osteoporosis (present vs. absent).

**Figure 2:**
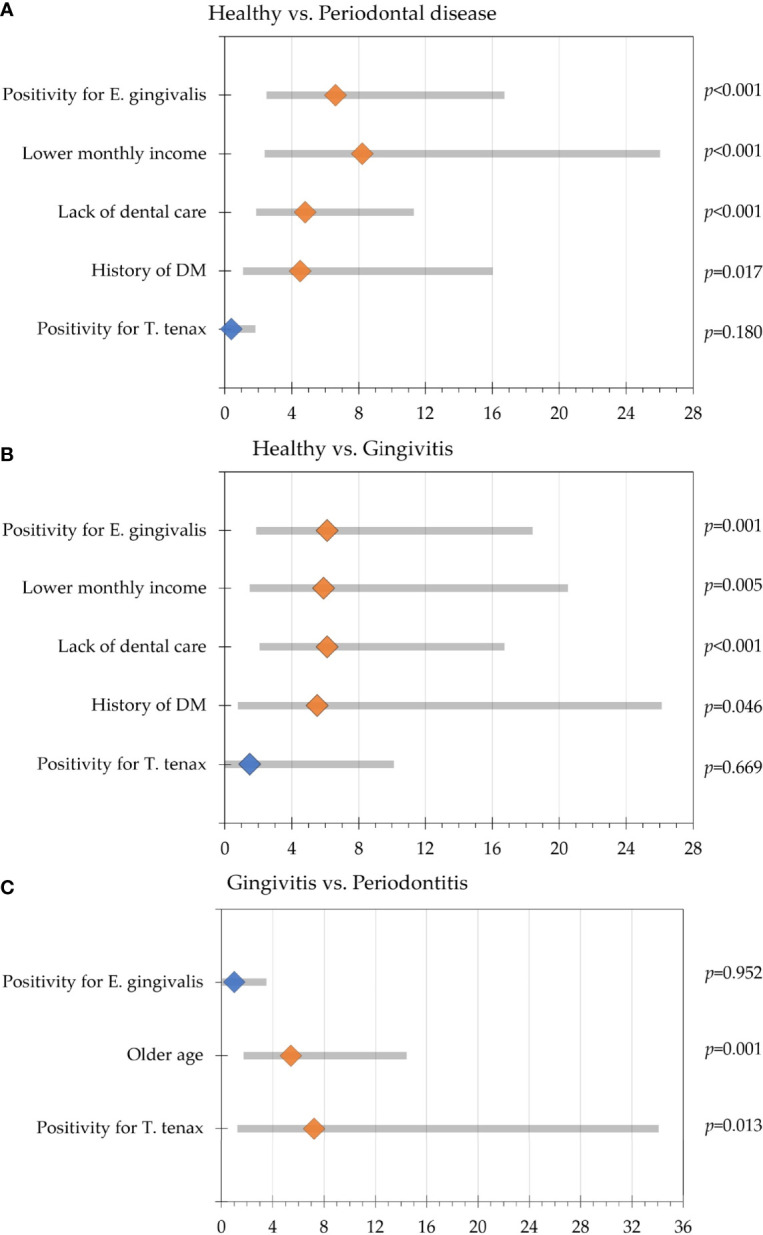
Multinomial regression analysis of participants’ variable association with periodontal disease. Odds ratios are represented by the diamond shapes (light orange for statistically significant result and blue color for statistically non-significant results), while the 95% confidence intervals are shown as the grey bars. **(A)** Comparison between the healthy group and the periodontal disease group (gingivitis and periodontitis); **(B)** Comparison between the healthy group and gingivitis group; **(C)** Comparison between the gingivitis and periodontitis groups.

Initial analysis was done with the dependent variable being health vs. periodontal disease and the presence/absence of oral parasites as the fixed factors. Positivity for *E. gingivalis* was correlated with the presence of periodontal disease with odds ratio (OR) of 6.6 (95% CI: 2.7 – 16.5; p<0.001), with the following covariates having significant correlation with the disease: lower monthly income (OR: 8.2, 95% CI: 2.6 – 25.8, p<0.001), the lack of dental care (OR: 4.8, 95% CI: 2.1 – 11.1, p<0.001), and history of DM (OR: 4.5, 95% CI: 1.3 – 15.8, p=0.017). Oral colonization by *T. tenax* was not found to be correlated with the presence of periodontal disease (p=0.180, **Figure 2A**).

Further analysis with dependent variable being health vs. gingivitis and the presence/absence of oral parasites as the fixed factors revealed that colonization by *E. gingivalis* was correlated with gingivitis (OR: 6.1, 95% CI: 2.1 – 18.2, p=0.001), while correlation with oral colonization by *T. tenax* lacked the statistical significance for upon comparing the healthy group with gingivitis (p=0.669). The covariates that were associated with gingivitis in relation to the healthy group were the lack of dental care (OR: 6.1, 95% CI: 2.3 – 16.5, p<0.001), lower monthly income (OR: 5.9, 95% CI: 1.7 – 20.3, p=0.005) and history of DM (OR: 5.5, 95% CI: 1.0 – 29.5, p=0.046, **Figure 2B**).

Comparison between the healthy and the periodontitis groups revealed that colonization by *E. gingivalis* and *T. tenax* were significantly correlated with periodontitis (OR: 6.4, 95% CI: 2.2 – 18.7, p=0.001 for *E. gingivalis*, and OR: 4.7, 95% CI: 1.0 – 21.8, p=0.045 for *T. tenax*). The covariates that were associated with periodontitis in relation to the healthy group were the lack of dental care (OR: 4.4, 95% CI: 1.6 – 11.7, p=0.003), lower monthly income (OR: 26.6, 95% CI: 2.8 – 249.7, p=0.004) and history of DM (OR: 4.9, 95% CI: 1.3 – 19.3, p=0.021).

Comparing the gingivitis and periodontitis groups revealed that *T. tenax* was significantly correlated with periodontitis (OR: 7.2, 95% CI: 1.5 – 33.8, p=0.013), while colonization by *E. gingivalis* lacked the statistical significance upon comparing the two groups (p=0.952). Besides colonization by *T. tenax*, older age was the only covariate to be correlated with periodontitis compared to gingivitis (OR: 5.4, 95% CI: 2.0 – 14.2, p=0.001, **Figure 2C**).

### Association of Oral Parasites With Periodontal Disease Extent

**Figure 3:**
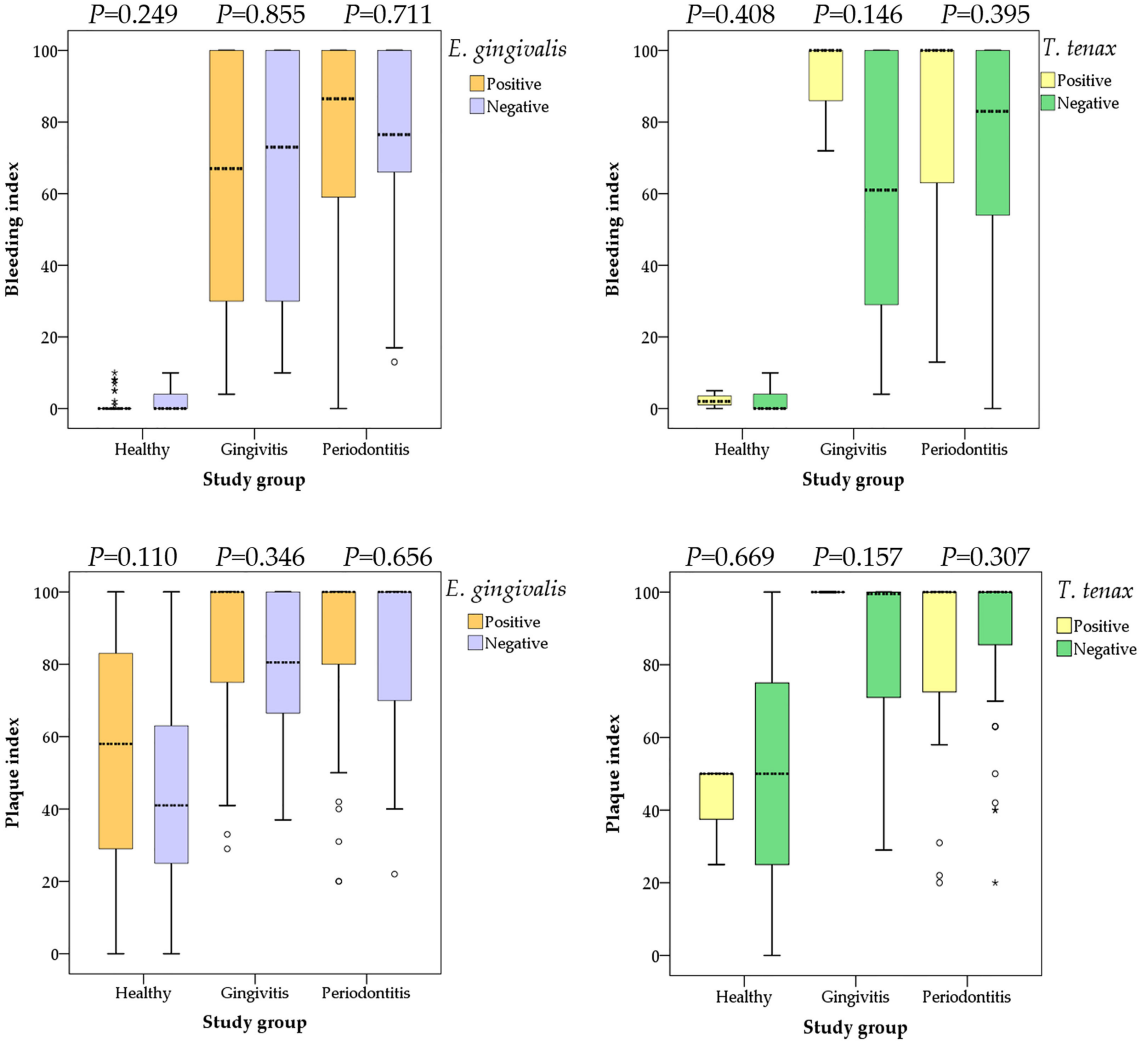
Comparison between the bleeding and plaque indices with colonization by oral parasites stratified by the study groups (health, gingivitis and periodontitis). P values were calculated using Mann Whitney *U* test. Median values are shown as dashed lines. Outlier values are shown as small circles, while extreme outlier values are shown as asterisks.

Data on the type of periodontal disease (localized vs. generalized) was available from 130 study subjects. The localized type comprised 18 individuals as opposed to 112 individuals with generalized periodontal disease. *T. tenax* was only detected among individuals with generalized periodontal disease compared to its total absence among those with localized periodontal disease (19.6% vs. 0.0%; p=0.039, χ^2^ test) and no dual colonization was detected either in the localized group compared to generalized group (p=0.051, χ^2^ test). Despite the higher prevalence of both oral parasites in individuals with advanced stage and grade of disease (as indicated by higher BI and PI), the differences lacked statistical significance as illustrated in (**Figure 3**).

### Estimation of the Co-Infection Rates by the Two Oral Parasites in the Study Sample

The concurrent detection of the two oral parasites was observed in 26 study subjects yielding a co-infection rate of 11.0% (95% CI: 7.6% – 15.6%). The co-infection rates were higher among periodontitis patients, individuals older than 38 years, non-Jordanians, participants with BMI > 25, participants with monthly income ≤ 1000 JOD, and those who reported the lack of any form of dental care (**Table 4**).Multinomial logistic regression analysis was conducted, with study group as the dependent variable, oral parasite status (co-infection vs. *E. gingivalis* only vs. *T. tenax* only vs. negative) as the fixed factor and the following covariates: age (> 38 years vs. ≤ 38 years, [38 years was the median age for the whole population]), sex (male vs. female), nationality (Jordanian vs. non-Jordanian), BMI (> 25 vs. ≤ 25), monthly income (≤ 1000 JOD vs. > 1000 JOD), dental care (no dental care vs. any form of dental care), smoking (current/ex-smoker vs. non-smoker), DM vs. non-diabetic, family history (present vs. absent), subjective evaluation of stress (more stressed if the score is 0-4 vs. less stressed if the score is 5-9), alcohol use (current/ex-user vs. non-consumer), osteoporosis (present vs. absent). Analysis showed the odds of periodontitis compared to the healthy group was the highest among individuals with coinfection (OR: 32.3, 95% CI: 4.3 – 236.2, p=0.001), followed by *E. gingivalis* only (OR: 6.5, 95% CI: 2.1 – 20.7, p=0.001), whereas the sole presence of *T. tenax* or negative result did not yield a statistically significant result (**Table 5**). On the other hand, co-infection by the two oral parasites did not yield significant correlations between the healthy vs. gingivitis groups or between gingivitis vs. periodontitis groups (**Table 5**).

**Table 4:** Factors associated co-infection by *Entamoeba gingivalis* and *Trichomonas tenax* in the study sample.

Characteristic	*Category*	*E. gingivalis* and *T. tenax* N^8^ (%)	*E. gingivalis* only N (%)	*T. tenax* only N (%)	Negative N (%)	P-value^9^
**Study group**	*Healthy*	2 (2.1)	43 (45.7)	1 (1.1)	48 (51.1)	<0.001
	*Gingivitis*	3 (5.7)	42 (79.2)	0 (0)	8 (15.1)	
	*Periodontitis*	21 (23.3)	59 (65.6)	2 (2.2)	8 (8.9)	
**Age**	*≤ 38*	7 (5.8)	70 (58.3)	2 (1.7)	41 (34.2)	0.012
	*> 38*	19 (16.4)	73 (62.9)	1 (0.9)	23 (19.8)	
**Sex**	*Male*	18 (14.8)	73 (59.8)	2 (1.6)	29 (23.8)	0.206
	*Female*	8 (7.0)	71 (61.7)	1 (0.9)	35 (30.4)	
**Nationality**	*Jordanian*	21 (9.5)	136 (61.3)	3 (1.4)	62 (27.9)	0.033
	*Non-Jordanian*	5 (33.3)	8 (53.3)	0 (0)	2 (13.3)	
**BMI^1^**	*≤ 25*	3 (2.9)	69 (65.7)	2 (1.9)	31 (29.5)	0.004
	*> 25*	23 (17.7)	74 (56.9)	1 (0.8)	32 (24.6)	
**Monthly income**	*≤ 1000 JOD^6^*	26 (14.0)	120 (64.5)	3 (1.6)	37 (19.9)	<0.001
	*> 1000 JOD*	0 (0)	23 (46.0)	0 (0)	27 (54.0)	
**Dental care level**	*Any form of care^7^*	7 (6.7)	54 (51.4)	1 (1.0)	43 (41.0)	<0.001
	*None*	19 (14.5)	89 (67.9)	2 (1.5)	21 (16.0)	
**DM^2^**	*No*	25 (12.1)	120 (58.3)	3 (1.5)	58 (28.2)	0.187
	*Yes*	1 (3.2)	24 (77.4)	0 (0)	6 (19.4)	
**Family history^3^**	*No*	20 (12.0)	97 (58.4)	2 (1.2)	47 (28.3)	0.700
	*Yes*	5 (9.3)	36 (66.7)	1 (1.9)	12 (22.2)	
**Smoking^4^**	*Never*	14 (10.7)	74 (56.5)	1 (0.8)	42 (32.1)	0.235
	*Current/ex-smoker*	12 (11.3)	70 (66.0)	2 (1.9)	22 (20.8)	
**Alcohol use**	*Never*	24 (10.6)	136 (60.2)	3 (1.3)	63 (27.9)	0.506
	*Current/former use*	2 (18.2)	8 (72.7)	0 (0)	1 (9.1)	
**Osteoporosis**	*No*	25 (11.7)	126 (59.2)	3 (1.4)	59 (27.7)	0.763
	*Yes*	1 (5.9)	12 (70.6)	0 (0)	4 (23.5)	
**Stress score^5^**	*Less stressed*	15 (9.9)	90 (59.6)	2 (1.3)	44 (29.1)	0.750
	*More stressed*	11 (12.8)	54 (62.8)	1 (1.2)	20 (23.3)	

^1^BMI, Body mass index; ^2^DM, History of diabetes mellitus; ^3^Family history, Previous diagnosis of periodontal disease in a family member; ^4^Smoking, Includes cigarettes, e-cigarettes, pipe, shisha, and narghile; ^5^Stress scale, Nine-item based scale that was adopted from the Perceived Stress Scale, to assess the stress-related factors with a range (0-9); ^6^JOD, Jordanian dinar; ^7^Any form of care, Any form of dental care including annual inspection, regular and irregular monitoring and cleaning; ^8^N, Number; ^9^P-value, Calculated using chi-squared test.

**Table 5:** Multinomial regression analysis assessing the correlation between oral parasite detection and disease states (healthy vs. gingivitis vs. periodontitis).

Variable	Periodontitis vs. Healthy		Gingivitis vs. Healthy		Periodontitis vs. Gingivitis	
	Odds ratio (95% CI^4^)	P-value	Odds ratio (95% CI)	P-value	Odds ratio (95% CI)	P-value
Co-infection^1^	31.961 (4.325–236.184)	0.001	4.841 (0.509–46.014)	0.170	6.602 (0.995–43.821)	0.051
*E. gingivalis*	6.543 (2.073–20.652)	0.001	5.684 (1.887–17.118)	0.002	1.151 (0.349–3.792)	0.817
*T. tenax*	5.03 (0.285–88.710)	0.270				
Reference (negative)						
**Covariates**						
Age	4.244 (1.404–12.828)	0.010	0.777 (0.248–2.434)	0.665	5.46 (2.059–14.478)	0.001
Sex	0.679 (0.247–1.871)	0.454	0.594 (0.221–1.599)	0.302	1.144 (0.438–2.985)	0.784
Nationality	0.319 (0.033–3.072)	0.323	0.909 (0.125–6.588)	0.925	0.351 (0.053–2.314)	0.277
BMI^2^	1.052 (0.371–2.986)	0.924	0.687 (0.251–1.878)	0.464	1.532 (0.608–3.861)	0.366
Monthly income	0.038 (0.004–0.358)	0.004	0.168 (0.049–0.58)	0.005	0.226 (0.022–2.356)	0.214
Dental care level	4.39 (1.641–11.747)	0.003	6.244 (2.310–16.878)	<0.001	0.703 (0.254–1.945)	0.498
DM^3^	0.202 (0.052–0.791)	0.022	0.184 (0.034–0.98)	0.047	1.101 (0.231–5.244)	0.904
Family history	0.454 (0.154–1.342)	0.153	1.173 (0.377–3.648)	0.783	0.387 (0.139–1.076)	0.069
Smoking	1.693 (0.628–4.563)	0.298	1.468 (0.558–3.861)	0.436	1.153 (0.457–2.910)	0.763
Alcohol use	0.382 (0.033–4.460)	0.443	0.576 (0.07–4.726)	0.607	0.664 (0.074–5.929)	0.714
Osteoporosis	0.72 (0.134–3.882)	0.702	0.254 (0.046–1.404)	0.116	2.837 (0.706–11.396)	0.142
Stress	2.58 (0.955–6.969)	0.062	1.463 (0.532–4.025)	0.461	1.764 (0.738–4.215)	0.202

^1^Co-infection, The concurrent detection of both Entamoeba gingivalis and Trichomonas tenax; ^2^BMI, Body mass index; ^3^DM, History of diabetes mellitus; ^4^CI, Confidence interval.

**Figure 4:**
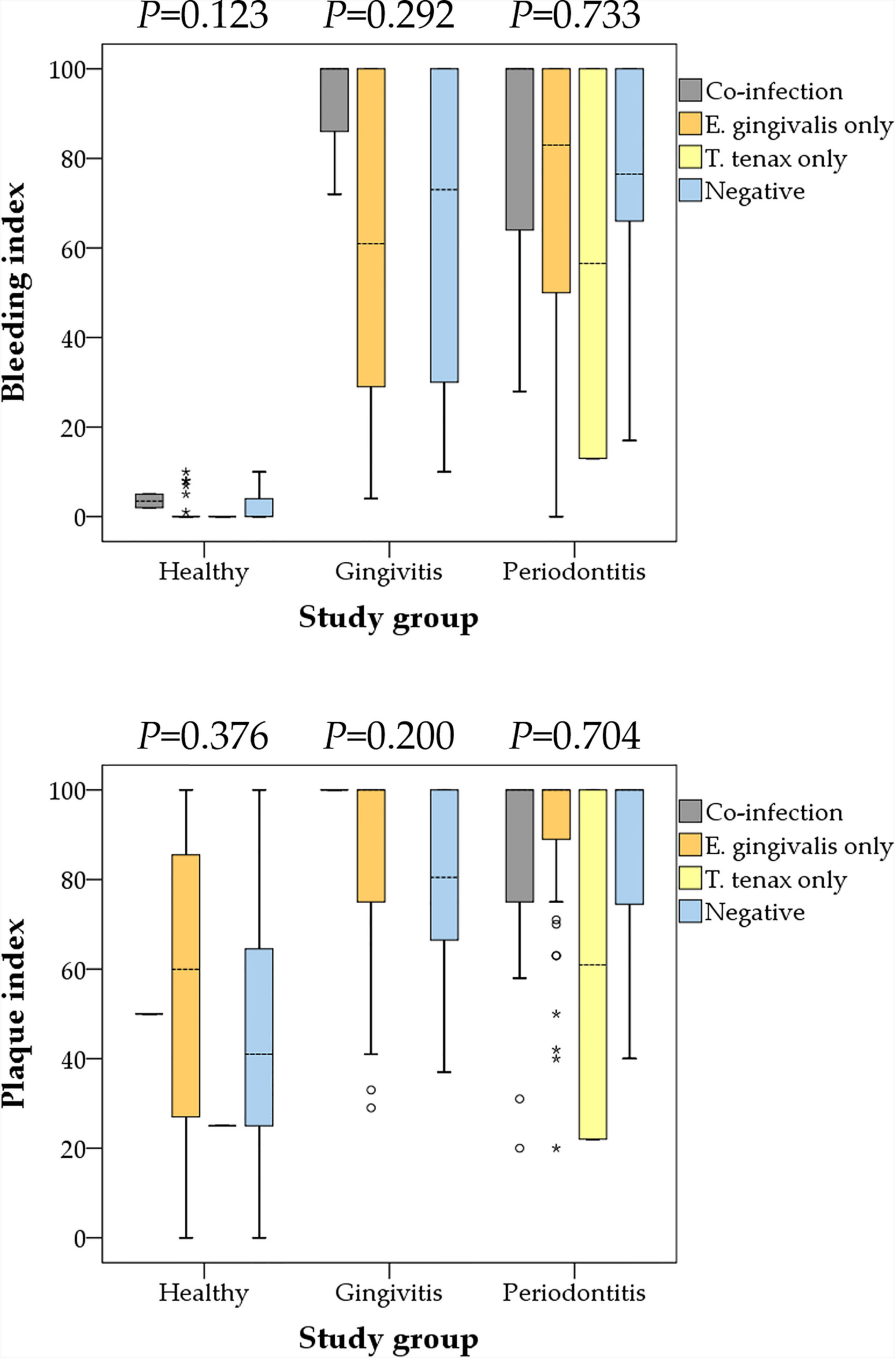
Comparison between the bleeding and plaque indices with oral parasite infection status stratified by the study groups (health, gingivitis and periodontitis). Co-infection denoted the concurrent detection of both *E. gingivalis* and *T. tenax*. P values were calculated using Kruskal Wallis test. Median values are shown as dashed lines. Outlier values are shown as small circles, while extreme outlier values are shown as asterisks.

Comparison of the BI and the PI among the three study groups (healthy vs. gingivitis vs. periodontitis) based on the oral parasite infection status (co-infection vs. *E. gingivalis* only vs. *T. tenax* only vs. negative result) did not yield any statistically significant differences (**Figure 4**).

## Discussion

Gingivitis and periodontitis are inflammatory conditions that can also be viewed as infectious diseases (Cekici et al., 2014). In periodontitis, the role of the bacterial fraction of the oral microbiome has been studied extensively with accumulating evidence pointing to its contribution to the etiology of the disease (Teles et al., 2013). However, the fraction of protozoa was not studied to a similar level compared to its bacterial counterpart (Deng et al., 2017; Bonner et al., 2018; Bisson et al., 2019; Santi-Rocca, 2020; Badri et al., 2021; Eslahi et al., 2021). Nevertheless, this protozoan fraction does not seem to be negligible in some clinical setups, with *E. gingivalis* RNA accounting for up to 9% of the total RNA in periodontal pockets (Deng et al., 2017). Thus, more studies are warranted to evaluate the potential role of the human oral “protozoome” in health and disease.

In the current study, we investigated the prevalence and risk factors for oral colonization by the currently known human oral parasites, *E. gingivalis* and *T. tenax*, for the first time in Jordan. The importance of this work is related to the following aspects: First, periodontal disease (gingivitis and periodontitis) has a high prevalence among individuals of different age groups, which poses significant risks to public health, including various associated conditions and potential tooth loss (Griffin et al., 2012; Kinane et al., 2017; Tonetti et al., 2017). Second, some key elements regarding the etiology and pathophysiology of periodontal disease have not been disentangled yet; hence, more research is needed to decipher these unresolved elements (Darveau, 2010; Hajishengallis, 2015; Lamont et al., 2018). Third, despite the uncertainties regarding the specific roles of different microorganisms in periodontal disease, the accumulating evidence points to conspicuous differences in the oral microbiome between health and disease, and the role of oral parasites in both states has not been clearly delineated yet (Santi-Rocca, 2020). Fourth, a few studies on the epidemiology of oral parasites originated from the Middle East and North Africa (MENA) region. A majority of these studies that were conducted in Egypt, Iran, Iraq and Saudi Arabia, have not relied on the molecular detection approach for estimating the burden of oral parasites (el Hayawan and Bayoumy, 1992; Athari et al., 2007; Ghabanchi et al., 2010; Ibrahim and Abbas, 2012; Ismail et al., 2017; Hassan et al., 2019).

Thus, we aimed to build on the previous work that had the same objective, while attempting to avoid some limitations of the previously published reports. For example, in this study, each sample was based on a mixture of saliva and dental plaques, as used for instance by (Bao et al., 2020), as opposed to relying on one of them solely for parasite detection, as used for instance by (Bonner et al., 2014). The advantage of this sampling approach is related to improving the sensitivity of detection as oral parasites can be found in either one of these sites (Rashidi Maybodi et al., 2016). Also, we relied on the reference molecular method currently applied for oral parasite detection, rather than relying on microscopic detection (Bonner et al., 2014; Santi-Rocca, 2020). In addition, we tried to improve the sensitivity of molecular detection in this study through using silica column-based DNA extraction method, which can help to remove PCR inhibitors, and we adopted sensitive PCR protocols that were previously validated (Kikuta et al., 1997; Schrader et al., 2012; Bonner et al., 2014).

The main result of the study was the finding of a profoundly higher prevalence of *E. gingivalis* (87.4%) among individuals with periodontal disease, compared to those in the healthy group (47.9%) using the PCR method. For *T. tenax*, the estimates were much lower and significant differences were found moving from 3.2% in the healthy group to 18.2% among individuals with periodontal disease. To assess the reproducibility of these results, a limited number of studies were found (Kikuta et al., 1996; Trim et al., 2011; Bonner et al., 2014; Garcia et al., 2018a; Santi-Rocca, 2020; Badri et al., 2021; Eslahi et al., 2021). The comparisons were further complicated by the reliance of a majority of the previous studies on microscopic detection methods (examination of wet mounts or permanent stained smears) (Athari et al., 2007; Ghabanchi et al., 2010; Al-hamiary et al., 2011; Ibrahim and Abbas, 2012; Yazar et al., 2016; Hassan et al., 2019). Possible explanations for discrepancy between the results of microscopic detection of oral parasites and the PCR-based results include: the subjectivity of the microscopic approach which depends on the skills and experience of the examiner, the number of the fields examined, the method of microscopy used (light vs. phase-contrast), the use of staining, the nature of mounting media, and the lag time between sampling and examination (particularly for wet mount examination which depends on the viability of oral parasites, since motility is one of the decisive defining features for diagnosis) as discussed previously by (Bonner et al., 2014).

Despite the variability in results of the previously published reports, two recurring patterns were observed and were in line with our results. First, the observation of an increase in the prevalence of both oral parasites moving from health to gingivitis and reaching the highest levels in periodontitis (Santi-Rocca, 2020; Badri et al., 2021). Second, the generally higher prevalence of *E. gingivalis* in comparison to *T. tenax* in both health and disease. Interestingly, a significant association between the presence of *T. tenax* and periodontal disease severity was also found which was manifested in its total absence in localized disease. This result should be interpreted with extreme caution considering the limited number of individuals with localized diseases that were included in the study (n=12). However, Marty et al. hinted to the potential existence of an association between oral colonization by *T. tenax* and severity of periodontal disease; thus, our observation might not appear as an unforeseen result (Marty et al., 2017; Bisson et al., 2019). Since the current consensus is the belief that the role of microbial communities rather than single microbes are implicated in the development of periodontal disease, it appears that the significant differences observed in this study among different groups and for the two oral parasites is genuine and the potential pathogenic roles of these oral parasites should be dissected continuously similar to recent work by Bao et al. (Mira et al., 2017; Bao et al., 2020).

In the few studies that used the molecular approach for identification of oral parasites, the prevalence of *E. gingivalis* was consistent with our results (Bonner et al., 2014; Garcia et al., 2018a; Dubar et al., 2020). It is important to note that the PCR primers used in the studies could be specific to the first *E. gingivalis* subtype, ST1; to the second subtype, ST2 or indistinctly detecting both subtype by real-time PCR (Trim et al., 2011; Bonner et al., 2014; Garcia et al., 2018a; Bao et al., 2020; Dubar et al., 2020).

Bonner et al. reported a slightly lower prevalence of 33.3% for *E. gingivalis* among healthy individuals, whereas Garcia et al. results were close at 48.6% for ST1. In the two other remaining studies, *E. gingivalis* was not detected at all among healthy individuals; however, these two studies suffered from two shortcomings. First, they used different sets of primers that might resulted in missing some cases particularly those with possible low parasite loads. Second, the two studies included smaller sample sizes (12 and 20 samples) (Kikuta et al., 1996; Trim et al., 2011). For periodontitis, results were available from few studies, and the prevalence of *E. gingivalis* ranged from 26.9% to 80.6% (Trim et al., 2011; Bonner et al., 2014; Garcia et al., 2018a). In a recent study by Bao et al, the frequency of *E. gingivalis* the healthy controls were lower compared to this study (15.0% vs. 47.9%) (Bao et al., 2020). However, the gap was smaller upon comparing the inflamed periodontal sites between the studies (77.0% vs. 87.4%). Possible explanation for the observed differences is a specificity linked to the included healthy participants or the use of different sampling approach: Bao et al. included all uninflamed areas at the buccal mucosa, hard palate, tongue, and the upper and lower dentitions (Bao et al., 2020). This points to the importance of reaching a consensus to unify the parasite detection approach between studies with similar aims that would help in further explorations and comparisons regarding the prevalence of oral parasites in different populations.

The few studies that used PCR for the detection of *T. tenax*, reported a higher prevalence among individuals with periodontitis compared to the control group (Athari et al., 2007; Mehr et al., 2015; Bisson et al., 2018; Bracamonte-Wolf et al., 2019). However, the overall prevalence of *T. tenax* in these studies varied considerably among the periodontitis patients (26.9% vs. 40.0% vs. 70.0%), which might be related to the differences in the studied populations.

Though gingivitis is well defined at the clinical level, its place in the pathophysiology of periodontitis has not been characterized yet, in particular at the microbiological level. As reviewed recently by (Santi-Rocca, 2020), *E. gingivalis* has been detected by PCR in gingivitis in only one study before, with a prevalence (81.3%) comparable to this in the periodontitis group (73.5%), and higher than in the healthy group (54.3%) (Garcia et al., 2018a). These results are consistent with the ones presented here (47.9% for health, 84.9% for gingivitis, and 88.9% for periodontitis) and in other studies using different methods (Santi-Rocca, 2020).

For *T. tenax*, the very low numbers of positive PCRs in the healthy and gingivitis groups (3/94 = 3.2% and 3/53 = 5.7%, respectively) do not allow to evidence differences among them, though they exist with the periodontitis patients (23/90 = 25.6%). Thus, our results suggest that gingival sulci in gingivitis *stricto sensu*, without bone destruction (excluding necrotizing ulcerative gingivitis), are infected by *E. gingivalis* and not by *T. tenax*. The difference in infection by *T. tenax* in patients with periodontitis may depend on other variables than the clinical parameters leading to classification in this category (e.g. the specific periodontal microbiota associated with periodontitis) (Benabdelkader et al., 2019).

Analysis of different individual variables for possible association with increased likelihood of harbouring the oral parasites was futile to say the least. Patterns in the whole population differed when analysis was done by stratification into the three individual groups, and also no specific patterns were consistently found in other studies (Albuquerque Júnior et al., 2011; Ibrahim and Abbas, 2012; Bracamonte-Wolf et al., 2019). However, an interesting observation that can be seen in this study is that colonization by oral parasites *per se* appeared to be an independent risk factor for periodontal disease. Indirect indicators of a lower socio-economic status (low income and absence of previous dental care) appeared to have the most obvious association with higher prevalence of oral parasites besides the increasing age irrespective of the individual group (which might be related to an increased likelihood of exposure). The known risk factors for periodontitis were not necessarily associated with higher prevalence of oral parasites, which makes us inclined to propose that the presence of oral parasites may not merely be a marker of the disease and might rather play a larger role that has not been appreciated yet.

Another finding of this study was description of a coinfection by both oral parasites among 11% of the study participants. The contemporaneous detection of *E. gingivalis* and *T. tenax* was evidently linked to a high correlation with periodontitis compared to the healthy individuals, with significant association with lack of dental care, lower monthly income, and older age. However, more studies are needed to elucidate the contribution of coinfection to periodontal disease. A previous study that was conducted among children in Mexico found a coinfection rate of only 1.3%, which was linked to poor dental hygiene (Cuevas et al., 2008).

Limitations of the current work were inevitable and included difficulty in matching different groups (health and disease), particularly for age and income levels, which precluded conducting the study in a case-control design. In addition, convenience sampling has the inherent limitation of potential bias, with the possibility that the recruited individuals may not be reflective of each study group. Moreover, the sampling approach that involved mixing the salivary and dental plaque specimens might result in underestimation of the potential correlations between oral parasites and the extent and severity of periodontal disease, and this should be considered in any future work studying such a potential correlation. The application of quantitative PCR could have resolved association between the parasite load and periodontal disease, especially for *E. gingivalis*, and this should be considered in any future work trying to link oral parasites in health and disease, especially with availability of an experimentally validated protocol for such an aim (Zaffino et al., 2019). Also, the strain variability particularly for *E. gingivalis* was not covered completely in this work since we did not use the ST2 primers aimed at the detection of the second currently known variant of *E. gingivalis* and this clear limitation should be considered by assessing the prevalence of the other strain in the future studies (Garcia et al., 2018a; Garcia et al., 2018b; Dubar et al., 2020). For the negative samples we did not rule out inhibition of PCR completely, as done by (Bonner et al., 2014), which can make our results an underestimation of the true prevalence.

## Conclusions

The higher prevalence of human oral parasites in periodontal disease compared to healthy individuals appears to be more than a mere marker for the disease and might also be associated with disease severity and potential for progression. Thus, the dogmatic view of these oral parasites as commensals needs to be re-evaluated and their role cannot be neglected in light of the results of this study that supplement the recent articles that pointed to similar links. However, the possible association between the oral parasites and periodontal disease severity should be addressed using longitudinal studies, besides the need for a refined sampling approach considering the site-specific nature of periodontal disease. It is recommended to conduct future studies with the same molecular approach since the sole use of microscopy can lead to significant underestimation of the true prevalence of these oral parasites. Future studies are needed to assess the molecular epidemiology of these oral parasites and to test whether variations in strains that do exist, have a significant contribution in health and disease (Cembranelli et al., 2013). The wide variability in *T. tenax* prevalence appeared to be existent in different geographic locations with different living standards and more studies are recommended to show if such variability is genuine, or if it is only a spurious correlation involving an underlying factor.

## Data Availability Statement

The raw data supporting the conclusions of this article will be made available by the authors, without undue reservation.

## Ethics Statement

The studies involving human participants were reviewed and approved by Institutional Review Board (IRB) at Jordan University Hospital (Ref. No. 239/2019). The patients/participants provided their written informed consent to participate in this study.

## Author Contributions

Conceptualization: AM, GŞ, JS-R, and MS. Data curation: MS. Formal analysis: MS. Investigation: AY, AM, DD, DT, AH, EA-F, YH, GŞ, JS-R, and MS. Methodology: AY, AM, DD, DT, AH, EA-F, YH, GŞ, JS-R, and MS. Funding acquisition: AM. Project administration: MS. Supervision: AM, JS-R, and MS. Visualization: MS. Writing - original draft: MS. Writing - review & editing: AY, AM, DD, DT, AH, EA-F, YH, GŞ, JS-R, and MS. All authors contributed to the article and approved the submitted version.

## Funding

This study was supported by funding from the Deanship of Academic Research at the University of Jordan with ref. No. (126/2019/19) granted on 30^th^ January 2019. The Deanship of Academic Research at the University of Jordan as the funding body, had no role in study design, data collection and analysis, decision to publish, or preparation of the manuscript.

## Conflict of Interest

JS-R is employed by Science and Healthcare for Oral Welfare,a private company that raises funds for research in science.

The remaining authors declare that the research was conducted in the absence of any commercial or financial relationships that could be construed as a potential conflict of interest.

## Publisher’s Note

All claims expressed in this article are solely those of the authors and do not necessarily represent those of their affiliated organizations, or those of the publisher, the editors and the reviewers. Any product that may be evaluated in this article, or claim that may be made by its manufacturer, is not guaranteed or endorsed by the publisher.
