# Supplementary material for: Oral Colonization by Entamoeba gingivalis and Trichomonas tenax: A PCR-Based Study in Health, Gingivitis, and Periodontitis
Source: Front Cell Infect Microbiol. 2021 Dec 7;11:782805. doi: 10.3389/fcimb.2021.782805 (PMC8688919; doi:10.3389/fcimb.2021.782805)
Supplement: Supplementary file 1 [file DataSheet_1.pdf]

frontiers

Supplementary Material

Supplementary Data

Study Subject Consent Form and Questionnaire

The University of Jordan

Department of Oral and Maxillofacial Surgery, Oral Medicine and Periodontology, Jordan University Hospital

Subject Information and Consent Form

To whom it might concern

I am the undersigned ……………………………, after explanation by my doctor, I agree willingly to participate and enter the research entitled:

"**The prevalence of oral parasites in Jordanian patients with chronic periodontitis: A prospective case-control study**"

I also agree to give the necessary samples to the research team and to adhere to the dates of follow-up visits. I authorize my doctor to give the necessary information about me to the study team for the necessary scientific uses, provided that my identity will remain unidentified and that my participation in the study does not affect the quality of the treatment I receive.

Name of the participant: …………………………….

Signature of the participant: …………………………….

Age/Date of Birth: …………………………….

Date: …………………………….

Witness: …………………………….

Thank you very much for agreeing to participate in this Research. The information provided by you in this questionnaire will be used for research purposes. It will not be used in a manner which would allow identification of your individual responses.

Age: ………………….

Gender:

Male

Female

If female, please check all that apply:

Pregnant

Nursing

Menopausal

Taking birth control pills

Marital Status:

Married

Single

Widow/widower

Divorced

Nationality:

Jordanian

Non-Jordanian

Body Mass Index (BMI):

Weight: ………………… kg. Height: ………………… cm.

Less than 20

20-25

25-30

More than 30

Monthly Income:

Less than 500 JD

500-1000 JD

More than 1000 JD

Dental Care Level:

None

Annual inspection

Regular monitoring and cleaning.

Irregular monitoring and cleaning.

Smoking:

Non-smoker

e-cigarettes

Pipe

Shisha, narghile

Smoker, ≤10 cigarettes daily

Smoker, >10 cigarettes daily

If smoker, please specify duration (in years) and quantity (in packs) of smoking:

Duration: ………………….

Quantity: ………………….

Diabetes:

No

Yes, latest HbA1c ≤7.0

Yes, latest HbA1c >7.0

Family History of Gum Disease:

Yes

No

N.A

Stress-related Factors (Tick the box if the answer is YES):

Are you bothered by feelings of loneliness?

Do you feel you are a member of a group of friends?

Do you know people to talk to?

Do you lack companionship?

Do you feel left out?

I feel that I am just as good as other people.

I feel hopeful about the future.

I am happy.

I enjoy life.

Alcohol Consumption:

None

Yes

If yes, please check all that apply:

Average number of beverages consumed:

1-7

More than 8

Alcoholic beverage most often consumed:

Beer

Wine

Liquor

Osteoporosis:

Yes

No

Thank You

Supplementary Methods

Classification of the Study Subjects

The diagnosis of gingivitis and periodontitis was based on diagnostic guidelines that were set by the 2018 new classification scheme for periodontal and peri-implant diseases and conditions (Caton *et al*., 2018).

The evaluation of study subjects started by inspecting the clinical appearance of the gingiva for any signs of inflammation including redness, loss of stippling and swelling, to assess the gingival health status for each participant. This was followed by the measurement of the following indices: periodontal screening and recording (PSR) index, bleeding index (BI) and plaque index (PI). Six teeth (maxillary right first molar, maxillary right lateral incisor, maxillary left first premolar, mandibular left first molar, mandibular left lateral incisor, and mandibular right first premolar) were used to calculate the PI and BI. Teeth were examined using the University of North Carolina (UNC) periodontal probe (15 mm) from four sites (buccal, mesial, distal, and lingual). Visible plaque was given a score of 1 and absence of plaque was given a score of zero with maximum score of 24/24 that was converted into percentage. Similarly, the BI was measured for the same teeth, but the probe was pushed with a light force into sulci and if bleeding was evident within 15 seconds then a score of 1 was recorded for that site.

For the PSR measurements, the dentition was divided into six sections (sextants); three for the upper arch and three for the lower arch (two posteriors and an anterior). The PSR for each sextant was measured by probing around all the teeth in the sextant and the most severe measurement was used to represent the sextant. The sextant was cancelled if there were less than two teeth present in that sextant. The PSR scores were recorded as follows: Code 0 was given to the sextant with no pockets, no calculus, no overhang restorations and no bleeding on probing, code 1 was given to the sextant with no pockets, no calculus, and no overhang restorations with bleeding on probing, code 2 was given to the sextant with no pockets, with presence of calculus and overhang restorations together with bleeding, code 3 was given to the sextant with pocket depth measuring between 3.5 mm and 5.5 mm, and code 4 was given to the sextant with pocket depths exceeding 5.5 mm. A star (*) was added in the presence furcation, mobility and recession 4mm or greater.

Following the aforementioned evaluation, the study subject was classified into the “healthy group” based on a BI < 10% and PSR score of zero, with no active pockets. The sample from each healthy participant was composed of a sub-gingival plaque along with saliva.

If the BI was ≥ 10% and ≤ 30%, then the participant was considered to have a “localized gingivitis”. The study subjects with BI > 30% were considered to have a “generalized gingivitis”.

A study subject having any sextant with PSR score of 3 or more was expected to be a periodontitis case and a full periodontal charting was performed. Full periodontal charting included measuring pocket depth, bleeding on probing, and recession of every tooth from six sites (the mesiobuccal, midbuccal, distobuccal, mesiolingual, midlingual, and distolingual). Invasion of the bifurcation and trifurcation of

multi-rooted teeth by periodontal disease was also recorded for each posterior tooth. Furcation invasion was done using Naber’s probe and then it was classified based on Hamp's classification.

Study subjects were classified into the “periodontitis group” after full periodontal examination if there was interdental clinical attachment loss detectable at ≥ two non-adjacent teeth, or buccal or oral clinical attachment loss (CAL) ≥ 3 mm with pocketing > 3mm detectable at ≥ two teeth. Once the patient was diagnosed with periodontitis, staging and grading were done according to the recent classification of 2018 (Caton *et al.*, 2018).

DNA Extraction and Amplification

Purification of DNA was done using QIAamp DNA Mini Kit (QIAGEN) according to manufacturer's instructions. Briefly, the saliva/dental plaque specimens were brought to room temperature and mixed well, followed by adding 20 µL of proteinase k to a total of 200 µL of the specimen. If the specimen volume was less than 200 µL, we added a proper volume of phosphate buffered saline to reach a final volume of 200 µL. This was followed by adding 200 µL of buffer AL to the sample/proteinase k and vortexing for until a homogenous solution was formed. The mixture was then incubated at 56 °C for ten minutes. After that, 200 µL absolute ethanol was added to the lysed sample a mixed by vortexing for 15 seconds followed by its transfer into QIAamp Mini spin column. This was followed by centrifugation at 6000 × g for one minute. Washing steps followed using buffers AW1 and AW2 and DNA elution was done using 200 µL of buffer AE and centrifugation at 6000 × g for one minute.

For the detection of oral parasites, two sets of PCR primers were used. For *E. gingivalis,* we used the same set of primers utilized by Bonner *et al*. with a minor modification of the reverse primer as follows: forward primer (5′-AGGAATGAACGGAACGTACA-3′) and reverse primer (5′-CCATTTCCTTCTTCTATTGTTTMAC-3′) with a product size of 203 bases (Bonner *et al*., 2014).

For *T. tenax,* we used the same set of primers utilized by Kikuta *et al*. as follows: PT3 forward primer (5′-AGTTCCATCGATGCCATTC-3′) and PT7 reverse primer (5′-GCATCTAAGGACTTAGACG-3′) with product size of 776 bases (Kikuta *et al*., 1997).

The PCR mix comprised 5 µL of the DNA eluate, 5 µL of 5×FIREPol Master Mix (Solis BioDyne), 1 µL of each primer and 13 µL of DNase/RNase free water. The steps of PCR were as follows: Initial denaturation for 3.5 minutes at 94 °C, 40 cycles of 1 minute at 94 °C for denaturation, 1 minute at 60 °C for primer annealing, 1 minute at 72 °C for elongation, a final elongation step for 5 minutes at 72 °C (Kikuta *et al*., 1997; Kucknoor *et al*., 2009; Bonner *et al*., 2014).

A volume of 6 µL of the final product was assessed using for 2% agarose gel electrophoresis for evaluation of the DNA product sizes. Proper positive and negative extraction and PCR controls were used to ensure the quality of DNA extraction and PCR and to rule out contamination. Positive controls were taken from periodontitis patients who were positive for E. gingivalis and T. tenax by microscopy and that yielded the correct band sizes, while the negative control was nuclease-free water. The housekeeping gene actin beta (ACTB) with accession number (NG_007992.1) was used to assess PCR inhibition of the sample and to ensure the efficiency of the DNA extraction procedure with the following primers: forward 5'-GTCCTGTGGCATCCACGAAA-3' and reverse 5'-AGTGAGGACCCTGGATGTGAC-3' and PCR product size of 265 bases.

Supplementary Results

Table S1. Detailed characteristics of the study subjects stratified based on the study group

Variable	Categories	Study group					
		Healthy		Gingivitis		Periodontitis	
		N[5]	%	N	%	N	%
Age group	< 20 years	1	1.1%	4	7.5%	2	2.2%
	20 - 29 years	54	57.4%	18	34.0%	11	12.2%
	30 - 39 years	14	14.9%	12	22.6%	10	11.1%
	40 - 49 years	11	11.7%	8	15.1%	28	31.1%
	50 - 59 years	8	8.5%	6	11.3%	33	36.7%
	60 years or more	6	6.4%	5	9.4%	6	6.7%
Sex	Male	40	42.6%	29	54.7%	53	58.9%
	Female	54	57.4%	24	45.3%	37	41.1%
Nationality	Jordanian	88	93.6%	48	90.6%	86	95.6%
	Non-Jordanian	6	6.4%	5	9.4%	4	4.4%
BMI[1]	< 20	9	9.7%	6	11.3%	6	6.7%
	20-25	41	44.1%	21	39.6%	22	24.7%
	25-30	25	26.9%	17	32.1%	29	32.6%
	> 30	18	19.4%	9	17.0%	32	36.0%
Monthly income	< 500 JOD[4]	15	16.0%	29	55.8%	70	77.8%
	500-1000 JOD	36	38.3%	17	32.7%	19	21.1%
	> 1000 JOD	43	45.7%	6	11.5%	1	1.1%
Dental care level	None	23	24.5%	37	71.2%	71	78.9%
	Annual inspection	6	6.4%	4	7.7%	3	3.3%
	Regular monitoring and cleaning	33	35.1%	3	5.8%	4	4.4%
	Irregular monitoring and cleaning	32	34.0%	8	15.4%	12	13.3%
Smoking	None	63	67.0%	26	49.1%	42	46.7%
	Smoker	30	31.9%	27	50.9%	45	50.0%
	Ex-smoker	1	1.1%	0	0.0%	3	3.3%
DM[2]	No	80	85.1%	50	94.3%	76	84.4%
	Yes, latest HbA1c ≤7.0	12	12.8%	2	3.8%	9	10.0%
	Yes, latest HbA1c >7.0	2	2.1%	1	1.9%	5	5.6%
Family history	Yes	19	21.6%	10	20.4%	25	30.1%
	No	69	78.4%	39	79.6%	58	69.9%
Alcohol	No	89	94.7%	51	96.2%	86	95.6%
	Yes	5	5.3%	1	1.9%	2	2.2%
	Ex-user	0	0.0%	1	1.9%	2	2.2%
OP[3]	Yes	4	4.3%	6	11.5%	7	8.1%
	No	88	95.7%	46	88.5%	79	91.9%

[1]BMI: Body mass index; [2]DM: Diabetes mellitus; [3]OP: Osteoporosis; [4]JOD: Jordanian dinar; [5]N: Number

References

Bonner, M., Amard, V., Bar-Pinatel, C., Charpentier, F., Chatard, J.M., Desmuyck, Y., Ihler, S., Rochet, J.P., Roux De La Tribouille, V., Saladin, L., Verdy, M., Girones, N., Fresno, M., and Santi-Rocca, J. (2014). Detection of the amoeba Entamoeba gingivalis in periodontal pockets. *Parasite* 21. doi:10.1051/parasite/2014029.

Caton, J.G., Armitage, G., Berglundh, T., Chapple, I.L.C., Jepsen, S., Kornman, K.S., Mealey, B.L., Papapanou, P.N., Sanz, M., and Tonetti, M.S. (2018). A new classification scheme for periodontal and peri-implant diseases and conditions - Introduction and key changes from the 1999 classification. *Journal of Periodontology* 89 Suppl 1**,** S1-S8. doi:10.1002/JPER.18-0157.

Kikuta, N., Yamamoto, A., Fukura, K., and Goto, N. (1997). Specific and sensitive detection of Trichomonas tenax by the polymerase chain reaction. *Letters in Applied Microbiology* 24**,** 193-197. doi:10.1046/j.1472-765x.1997.00379.x.

Kucknoor, A.S., Mundodi, V., and Alderete, J. (2009). Genetic identity and differential gene expression between Trichomonas vaginalis and Trichomonas tenax. *BMC Microbiology* 9**,** 58. doi:10.1186/1471-2180-9-58.
